# The effect of sampling methods on the validity and reliability of the estimation of the orbital stability of human gait

**DOI:** 10.1098/rsos.250106

**Published:** 2025-08-13

**Authors:** Jeongin Moon, Jooeun Ahn

**Affiliations:** ^1^ Department of Physical Education, Seoul National University, Seoul, Republic of Korea; ^2^ Soft Robot Research Center, Seoul National University, Seoul, Republic of Korea; ^3^ Institute of Sport Science, Seoul National University, Seoul, Republic of Korea

**Keywords:** Floquet multiplier, gait orbital stability, sampling conditions, bias and variance reduction, stochastic linear model

## Abstract

Floquet multiplier (FM) is a commonly used metric for evaluating gait orbital stability in biomechanics. However, variability of human gait and noise from various sources can induce significant bias and variance in the estimation of FM. Furthermore, FM is employed in gait analysis without standardized protocols, leading to highly case-dependent outcomes. To address these challenges, we quantify the effects of sampling conditions on the accuracy and consistency of FM estimations. We recruited 20 healthy participants and conducted five trials of 10 minutes of walking per participant. Using individualized Jacobian matrices calculated from the walking experiments, we synthesized multiple sets of virtual time series with varying lengths and trial counts. Using stochastic linear models, we simulated the error dynamics depending on the sampling methods. The bias and variance of FM estimates decreased as the time series lengthened, achieving a strong correlation with the true value after 140 strides for 14-dimensional state vector. Our results further suggest that partitioning a long time series into appropriately sized segments can yield more reliable FM estimates, reducing both bias and variance in FM estimations.

## 1. Introduction

Stability is an essential component of functional gait [1], but it often diminishes with ageing or the onset of chronic diseases, which increases susceptibility to falls [2,3]. For proper diagnosis of weakening of stability and implementation of adequate intervention, accurate and reliable assessment of stability is critical. However, conventional criteria for assessing gait stability, such as variability in stride time or step width, often fail to capture subtle differences in gait stability [4–6]. As an alternative, Floquet multiplier (FM) provides a more comprehensive evaluation of gait stability by directly quantifying a system’s ability to return to a stable orbit after disturbances [7].

Multiple studies have demonstrated the efficacy of FM in analysing gait stability across various conditions and populations. Hurmuzlu *et al.* quantified orbital stability of gait using FM and showed that the FM of healthy individuals is usually lower than that of patients [8]. Additionally, Dingwell *et al.* demonstrated the impact of sensory deficit on maintaining the orbital stability of gait [9]. Furthermore, the strategic difference in running between novice and the professional runners could also be quantified using orbital stability [10]. These studies demonstrate that FM can be a practical metric for capturing the dynamic properties of orbital stability in human locomotion.

Although FM appears to be useful for analysing gait stability, obtaining FM involves significant challenges. The results of FM estimation are highly dependent on parameter settings, such as the dimension of state space, the point of the Poincaré section in the limit cycle and the sampling condition [11]. These variations in parameter settings across studies have led to inconsistent estimates of gait orbital stability indices, thereby limiting the practical applicability of FM [10,12–15]. Riva *et al.* demonstrated that the minimum number of strides required to obtain reliable FM estimates is larger than the conventional choice, and if this condition is not met, FM estimation can exhibit very poor reliability [16].

In response to these challenges, numerous studies have sought to refine methods for evaluating FM. Ahn & Hogan examined the influence of noise and time series length on bias in FM estimation, proposing a noise-robust algorithm applicable in cases where the state is defined as a scalar [17]. In addition, Lee *et al.* proposed that multiple measurements are necessary to ensure reliable FM estimation [18]. Furthermore, Dingwell & Cusumano demonstrated that data surrogation can improve the validity of FM estimations by distinguishing deterministic components from noise [19]. It is widely recognized that an adequate sample size is essential for accurately capturing the structural patterns within the data [16]. Nonetheless, the sampling criteria necessary for achieving accurate and consistent FM values remain unaddressed.

In this study, we aim to comprehensively quantify the effects of data sampling methods on the accuracy and consistency of FM estimations. To this end, we collected experimental data from actual human walking trials and conducted simulations to examine the validity and reliability of FM estimations across a broad range of sampling conditions. We hypothesized that the validity and reliability of FM estimation could be significantly enhanced not only by the length of continuous time series but also by the number of trials. Given that data collection is always limited due to subject availability and capability of the participants, establishing concrete data sampling guidelines for FM estimation can reduce the uncertainty in gait stability measures and enable more reliable stability assessments within limited data acquisition periods.

## 2. Material and methods

### 2.1. Participants

Twenty healthy young adults (19 males, 1 female; age: 25 ± 4.5 years; height: 176.8 ± 4.6 cm; mass: 73.0 ± 5.0 kg) participated in the study. All participants were free from symptoms that could alter gait and reported no known orthopaedic, cardiovascular or neuromuscular impairments. Prior studies informed the selection of an effect size of 0.47 [7]. With this effect size, G*Power software calculated the sample size as 20 to achieve a significance level of 0.05 and a power of 0.95 [20].

### 2.2. Equipment

The instrumented treadmill (Bertec, USA; belt length: 2.5m; belt width: 1.2m) was utilized for the level-walking task. The participants’ gait kinematics were recorded using a motion capture system (Arqus, A5, Qualisys, Sweden) with lower-body marker sets (Rizzoli) at a sampling frequency of 100 Hz [21].

### 2.3. Walking experiment

Prior to data collection, participants donned comfortable walking attire and engaged in a 5 min treadmill warm-up to acclimatize to the walking environment [22]. Static posture was recorded for 3s to establish the joints' initial Cardan angles for subsequent analysis. All participants performed five independent 10 min level walking tasks at 1.17 m s^−1^ on a treadmill, with 5 min rest intervals provided between each trial. To minimized variability associated with self-paced speed, we employed a fixed walking speed based on the Froude number, consulting a previous study on biomechanics of walking [23]: $V=0.4\sqrt{gl}$, where *g* is gravitational acceleration (9.81 ms^−2^) and *l* is the average leg length (0.87 m) obtained from Korean anthropometric data [24].

### 2.4. Simulation procedure

**Figure 1.**
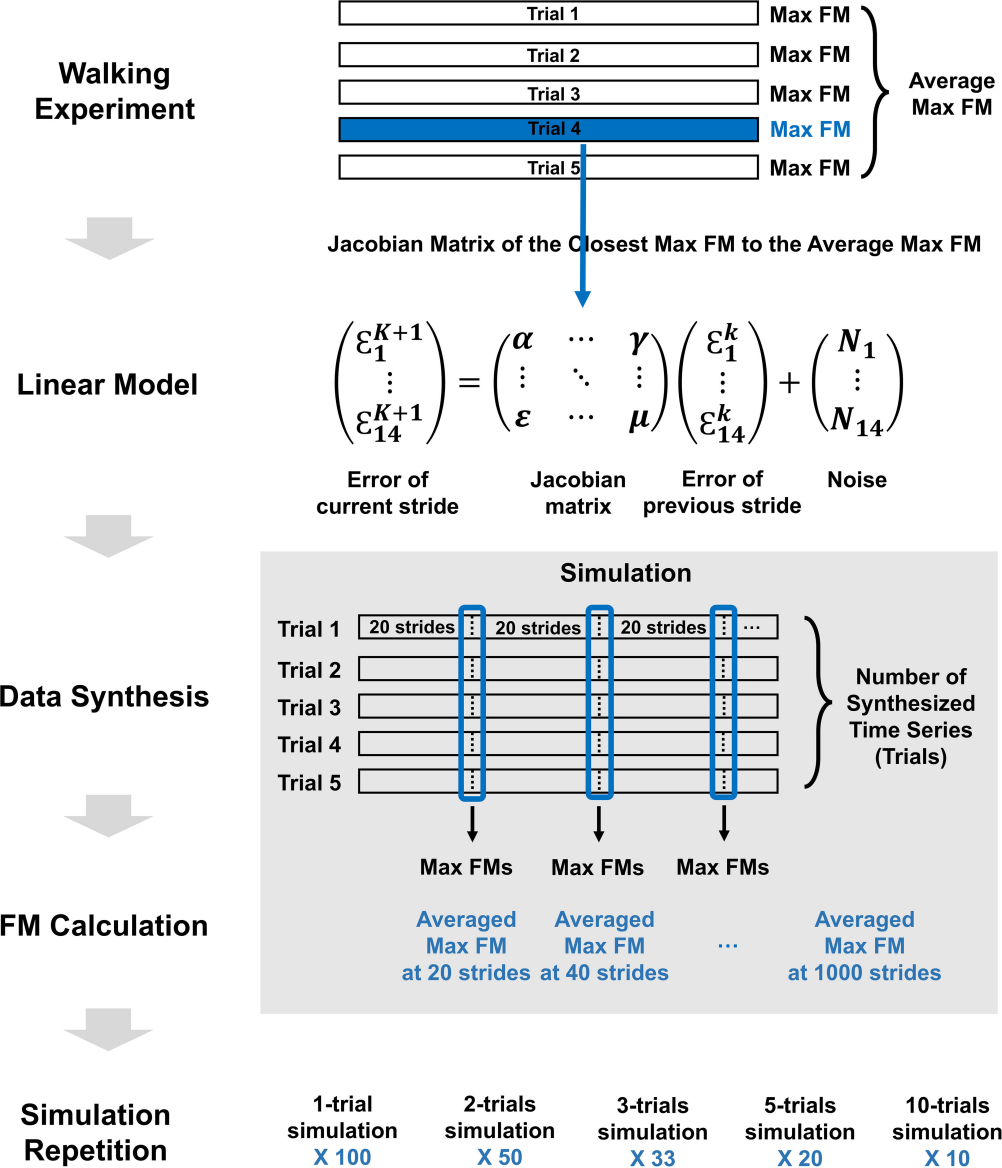
The scheme of simulation. The individualized Jacobian matrix for each subject was selected as the matrix of which the maximum eigenvalue was closest to the average maximum FM obtained from the walking experiment. The number of simulation repetitions was determined by the trial count so that the total number of generated trials was 100 or 99.

To evaluate the effect of the number of strides and trials on the accuracy and consistency of FM estimates, we conducted a simulation study utilizing the information obtained from the walking experiment. The simulation procedure is illustrated in figure 1 and involves the following steps:

(1) *14-dimensional state vectors*: The kinematics of the lower body was described using a 14-dimensional state vector representing the posture at each instant of left heel strike: $\vec{S}=(\theta_{lhip_{x}},\;\theta_{lhip_{y}},\theta_{lhip_{z}},\theta_{lknee_{x}},\theta_{lankle_{x}},\theta_{lankle_{y}},\theta_{lankle_{z}},\theta_{rhip_{x}},\;$$\theta_{rhip_{y}},\theta_{rhip_{z}}\theta_{rknee_{x}},\theta_{rankle_{x}},\theta_{rankle_{y}},\theta_{rankle_{z}})$. State vectors from consecutive strides constituted a 14-dimensional multivariate time series.
(2) *FM calculation from walking experiment*: The time-series data for each trial were mean-centred by subtracting the trial’s mean value to achieve a zero-mean signal. The Jacobian matrix and the maximum FM for each trial were then calculated using the ordinary least squares method [7].
(3) *Individualized Jacobian matrices*: Among the five Jacobian matrices calculated from the walking experiment, the matrix whose maximum FM was closest to the average of the five maximum FMs obtained from the experiment was selected as the representative individualized Jacobian matrix for each participant.
(4) *Linear Gaussian model*: We synthesized a discrete time series of state vectors using a stochastic linear model [25]. To capture the intrinsic variability of the original signal, we utilized the representative Jacobian matrix and the covariance structure from the walking experiment trials. Specifically, independent multivariate Gaussian noise with zero mean and the specified covariance matrix of the trial was introduced, consulting a widely recognized method in time-series analysis and machine learning [26,27].
(5) *Time series synthesis*: Each time series consisted of 1000 time points, corresponding to 1000 consecutive strides of heel strike motion. Each trial time series was synthesized independently with a randomly initialized state.
(6) *FM calculation and averaging*: FMs were calculated every 20 strides within each time series, starting from the 20th stride up to the 1000th stride. For participants with multiple trials, the maximum FMs calculated from trials of the same data length were averaged.

### 2.5. Data processing

#### 2.5.1. Three-dimensional Cardan angles of lower-body joints

We calculated the three-dimensional Cardan angle of the hip, knee and ankle using the human body modelling software Visual 3D (C-Motion, USA). The coordinate system of the joint angle follows the default Cardan sequence of the software: X (flexion/extension), Y (abduction/adduction) and Z (internal rotation/external rotation), which signify movements in the sagittal, frontal and transverse planes, respectively [28].

#### 2.5.2. The state space

**Figure 2.**
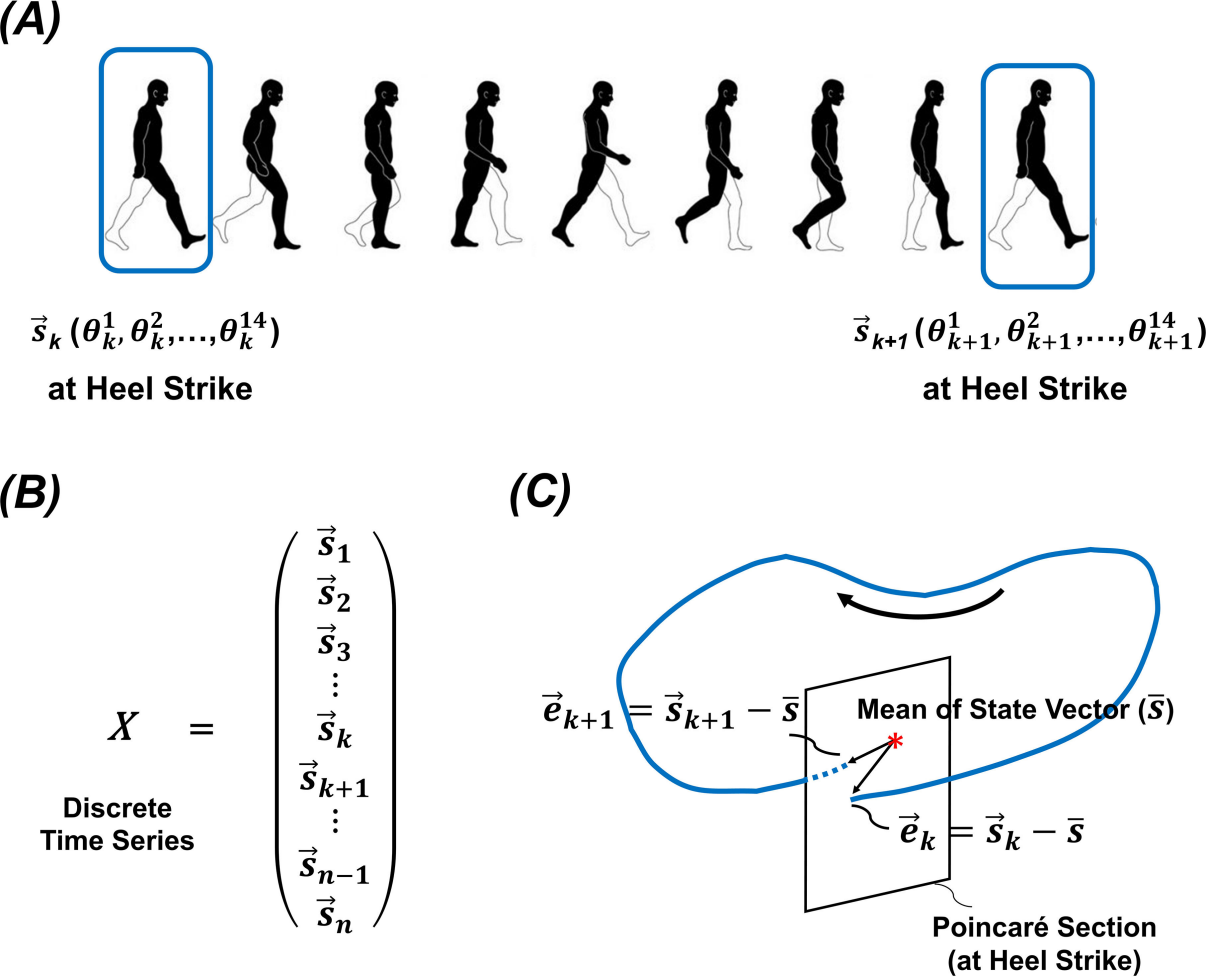
The definition and representation of state vector. (*A*) The state vector is defined using 14 lower body joint angles, including three-dimensional angles of the hip, flexion/extension of the knee and three-dimensional angles of the ankle for both limbs, recorded at the heel strike. (*B*) A discrete time series of gait data are used to calculate FM. (*C*) The Poincaré section is defined as the left heel strike instant (0% point of normalized stride), and the deviations from the mean state vector are defined as the error vectors.

We defined the left heel strike event as the instant when the heel marker was furthest from the pelvis centre of mass in the anterior direction and used this event to segment individual strides [29]. Subsequently, the joint angles of the lower body at the left heel strike instants were compiled to construct discrete time series, and 14 joint angles of the lower body joints (three-dimensional angles of the hip; flexion/extension of the knee; and three-dimensional angles of the ankle of both limbs) constructed a state vector, as shown in figure 2. Since the limit cycle of the human gait system is unknown, we used the mean of the time series as a proxy for the system’s limit cycle [30]. The deviation from this mean state vector was defined as the error vector for each stride.

#### 2.5.3. Mathematical representation of a return map

Although the transition function governing the state within the periodic gait cycle is nonlinear, we can approximate the stride-to-stride dynamics using the first-order Taylor expansion equation (2.1), under the assumption of small perturbations [31,32]. This approach linearizes a nonlinear system in a small neighbourhood around a fixed point:


(2.1) $${\vec{s}}_{k+1}-{\vec{s}}^{*}=\;J\cdot \left({\vec{s}}_{k}-{\vec{s}}^{*}\right)+\Delta_{k+1}\;\;\text{or}\;{\vec{e}}_{k+1}=\;J\cdot {\vec{e}}_{k}+\Delta_{k+1}\;,$$

where $\vec{s_{k}}$ is the state at *k*^th^ stride, $\vec{s}$^*^ is defined as the mean of states, ${\vec{e}}_{k}$ is the error at *k*^th^ stride which is equivalent to ${\vec{s}}_{k}-{\vec{s}}^{*}$, *J* is the Jacobian matrix of relating ${\vec{e}}_{k}$ and ${\vec{e}}_{k+1}$ and ${∆}_{k+1}$ is Gaussian noise added in each cycle as a random variable. In this framework, assessing orbital stability involves identifying the optimal *J* that best characterizes the linear relationship between consecutive errors.

#### 2.5.4. Ordinary least squares method for autoregressive processes

Estimating the error vector of next state using the error vector of current state can be described as an mutivariate autoregressive process [33]. Specifically, autoregressive process of order one is equivalent to the ordinary least squares (OLS) algorithm [18]. All elements of the error vector from the current state are used as independent variables, and elements of the error vector from the next state are used as dependent variables. In general, the normal equation is utilized to determine the coefficients in multiple regression analysis:


(2.2) β→^=argmin(y→−Xβ→)T(y→−Xβ→)β→^=(XTX)−1XTy→,  var(β→^)= (XTX)−1σ2,

where *X* is the time series matrix consisting of *m* independent variables as columns*,* $\vec{y}$ is the time series of the dependent variable and $\hat{\vec{\beta }}$ represents the estimated coefficients for the *m* independent variables. In summary, the Jacobian matrix, which consists of the estimated coefficients for a multivariate autocorrelation system, can be simplified as follows:


(2.3) $$\hat{J}={\left(\sum_{k=1}^{n-1}{\vec{e}}_{k}^{T}{\vec{e}}_{k}\right)}^{-1}\left(\sum_{k=1}^{n-1}{\vec{e}}_{k}^{T}{\vec{e}}_{k+1}\right),$$

where ${\vec{e}}_{k}$ is a 1×m error vector calculated as ${\vec{s}}_{k}-\overset{-}{s}$, *n* is the number of data points and the resulting $\hat{J}$ is an m×m matrix.

#### 2.5.5. Maximum eigenvalue of the Jacobian matrix

FMs are the eigenvalues of the estimated Jacobian matrix. In this study, as the state of the system is represented by a set of joint angles, each eigenvector ($\vec{\nu }$) associated with an eigenvalue (λ) corresponds to a specific kinematic mode of the system. The system is considered asymptotically stable if all eigenvalues of the Jacobian matrix have magnitudes less than unity [7]. Conversely, if any eigenvalue has a magnitude greater than unity, the system becomes unstable over consecutive strides due to the divergence of error in the associated kinematic mode, regardless of stability in other modes [33]. Therefore, the maximum FM is used as an index of orbital stability.

#### 2.5.6. Condition number

The condition number is a metric used to detect multicollinearity, which occurs when independent variables are highly correlated. It is defined as the ratio of the largest singular value of a design matrix to the smallest singular value:


(2.4) $$\kappa \left(X\right)=\frac{\sigma_{max}}{\sigma_{mi\mathrm{n,}}}$$

where $\sigma_{max}$ is the largest singular value and $\sigma_{min}$ is the smallest singular value of the matrix X. The condition number less than 10 indicates little to no multicollinearity among the independent variables. The condition number between 10 and 30 indicates moderate multicollinearity, meaning that some independent variables are correlated. When κ reaches 30 or higher, it signifies severe multicollinearity, which can result in unreliable and unstable regression estimates [34].

#### 2.5.7. Statistical analysis

For evaluating the validity and the reliability of the FM estimation, we conducted several types of statistical analysis. First, a linear mixed model (LMM) was employed to assess the main effects of the number of strides and the number of trials, as well as their interaction, on the FM estimates, which were log-transformed to meet normality, skewness and kurtosis criteria for the analysis. Second, the accuracy of FM estimates was quantified using the Pearson correlation. The true FMs of individualized Jacobian matrices and what were estimated using the simulated time series were compared using the 20 individualized datasets in different data sampling conditions. A Pearson’s *r* value greater than 0.7 was regarded as a sign of strong correlation [35]. Third, the consistency of FM measurements was calculated using a two-way random effects model of intra-class correlation (ICC2). For this, both the subjects and raters (or measurement sessions) are treated as random factors, implying that each rater and subject is randomly sampled from a larger population. An ICC2 coefficient value greater than 0.75 was regarded as a sign of good reliability [36]. Finally, the discrepancy between FM estimates from the walking experiment and the simulation across varying numbers of strides was analysed using a one-way repeated measures ANOVA. Pairwise comparisons were conducted using the least significant difference post-hoc test. The level of statistical significance was set at *p* < 0.05.

## 3. Results

### 3.1. The dependency of FM estimates on both the number of strides and the number of trials in the simulation

**Figure 3.**
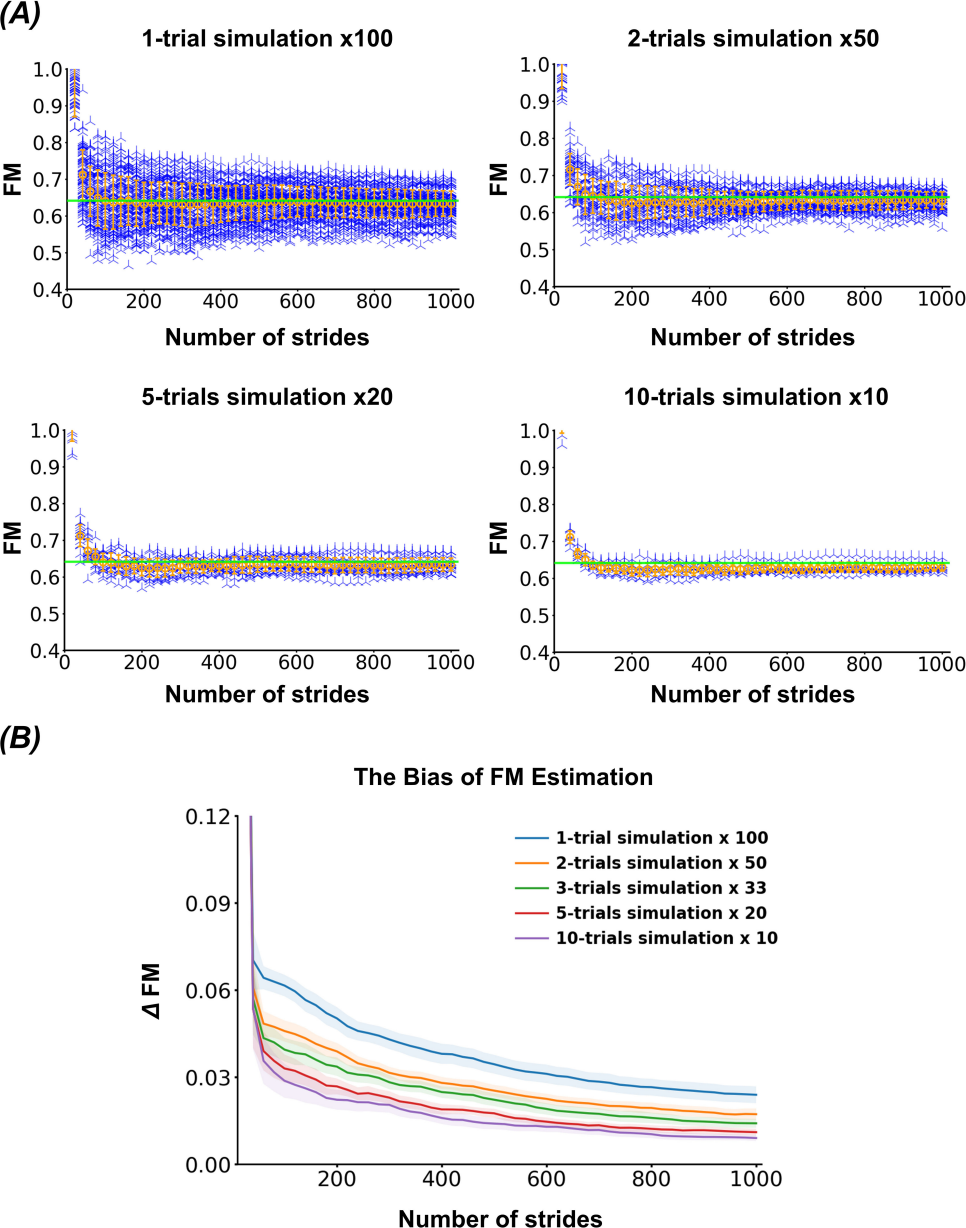
Effects of data sampling on FM estimation. (*A*) Representative FM estimates from synthetic datasets based on the individualized Jacobian matrix of Subject 1. Each plotted point represents an FM value estimated under different data sampling conditions, varying in the number of strides and the number of trials. The green horizontal line in each plot indicates the true maximal FM value obtained from the individualized Jacobian matrix. (*B*) The average bias of FM estimates calculated from 20 individualized synthetic datasets under different sampling conditions. The mean and 95% confidence intervals across repetitions are shown as a line and shaded area, respectively.

An LMM revealed significant fixed effects of both the number of strides (F(49, 4731) = 564.02, *p* < 0.001) and the number of trials (F(4, 4731) = 2383.25, *p* < 0.001) on the bias of estimated FMs, as shown in figure 3. Additionally, a significant interaction between strides and trials was observed (F(196, 4731) = 1.68, *p* < 0.001), indicating that the effect of the number of strides on bias depends on the number of trials. The log-transformed FM values exhibited skewness (1.21) and kurtosis (4.08) in the data distribution.

### 3.2. The evaluation of the accuracy and the consistency of the FM estimates in the simulation

**Figure 4.**
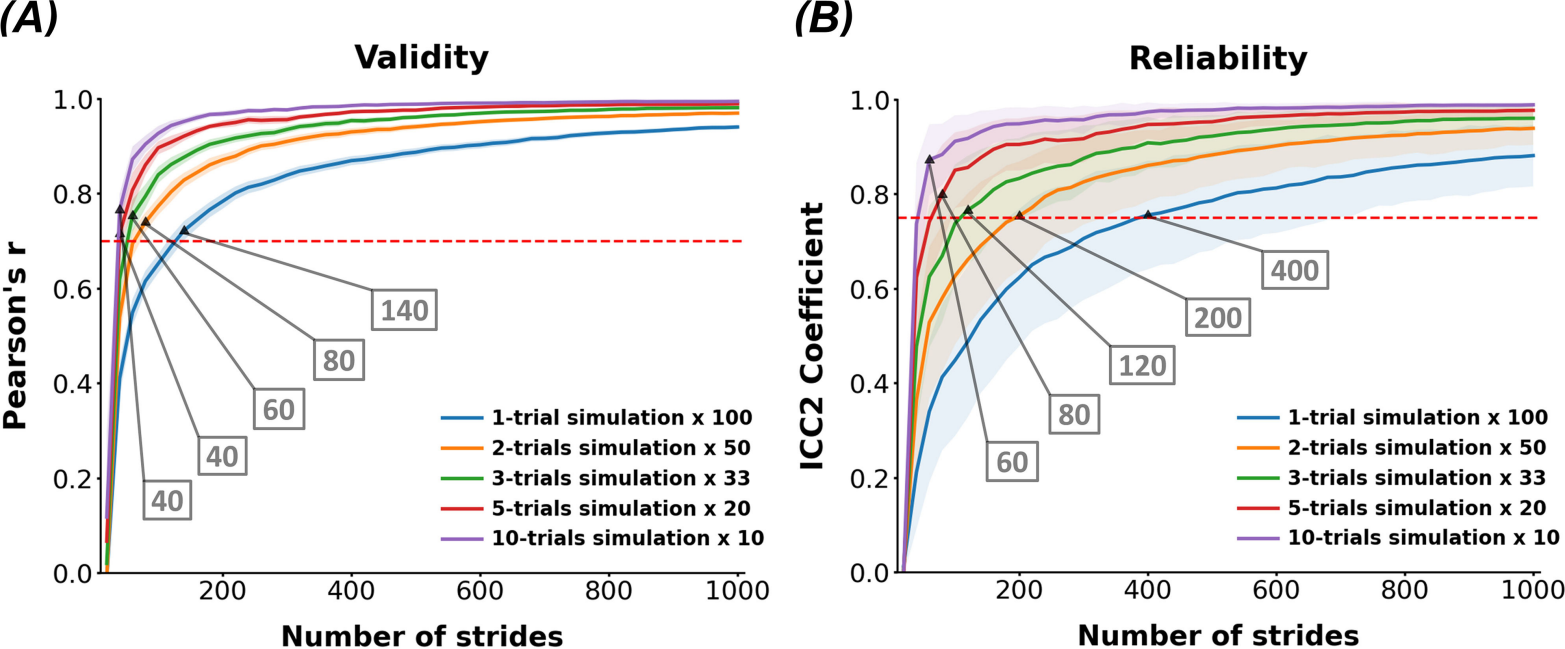
Validity and reliability of FM estimation under varying data sampling conditions. (*A*) Pearson correlation coefficients (Pearson’s *r*) between the FMs from individualized Jacobian matrices and what were estimated using the synthetic datasets across various data sampling conditions. (*B*) Intraclass correlation coefficients (ICC2) for both random subjects and raters (iterations), calculated using 20 synthetic datasets under varying data sampling conditions. The mean and 95% confidence intervals for FM estimates are represented by a line and shaded area, respectively. The red line indicates the threshold for a strong correlation level (0.7) for validity and a good level of agreement (0.75) for reliability. The numbers in the boxes represent the necessary strides for the validity or reliability to exceed the threshold when the increment of the used strides was 20.

The Pearson correlation coefficient was calculated to compare the FMs from individualized Jacobian matrices and those estimated using the simulated time series under various data sampling conditions. As shown in figure 4*A*, the mean discrepancy between the two decreases as the number of strides and the number of trials increase. The required length of strides for the correlation coefficient to reach the level of strong correlation (0.7) was 140, 80, 60, 40 and 40 strides when the number of trials was 1, 2, 3, 5 and 10, respectively.

The ICC2 was calculated using 20 individual synthetic datasets under various data sampling conditions. As shown in figure 4*B*, the consistency of the estimated FMs increases as the number of strides and the number of trials increase. The required length of strides for the ICC2 to reach the level of good agreement (0.75) was 400, 200, 120, 80 and 60 strides when the number of trials averaged was 1, 2, 3, 5 and 10, respectively.

### 3.3. The comparison between FM estimates from walking experiments and those from simulation

**Figure 5.**
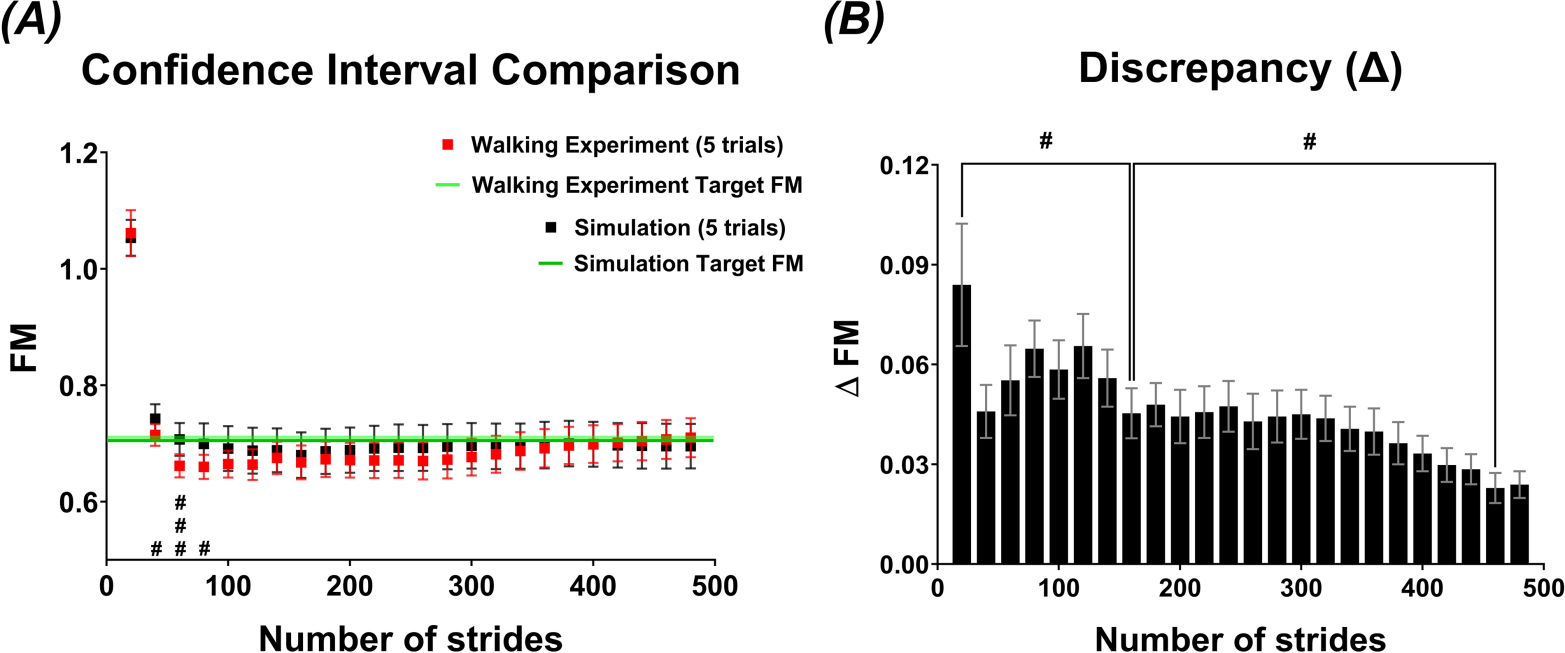
Discrepancies in FM estimates between walking experiment and simulation. (*A*) Mean and 95% confidence intervals of FM estimates for 20 subjects in both the walking experiment and simulation under various data sampling conditions. The confidence intervals of the walking experiment (red) and simulation (black) show increased overlap as the number of strides increases. (*B*) Mean and standard error of the discrepancies (ΔFM) between FM estimates from the walking experiment and simulation. The discrepancies significantly decrease as the number of strides increases. Both the walking experiment and simulation were conducted with five trials and a maximum of 500 strides to ensure comparability (#: *p* < 0.05, ##: *p* < 0.01, ###: *p* < 0.001).

An independent *t*‐test showed significant differences between the FM estimates from the walking experiment and the simulation when the number of strides was below 100 (figure 5*A*). A one-way repeated measures ANOVA revealed that the discrepancy between the two values decreased significantly as the number of strides increased (F(23, 437) = 3.67, *p* < 0.001) (figure 5*B*).

### 3.4. Multicollinearity among the predictable variables

The average and standard error of the condition number obtained from the walking experiment data of 20 subjects were 12.2 and 0.994, respectively. All the condition numbers were below 30, and some were below 10, suggesting low to moderate multicollinearity among lower body joint angles.

## 4. Discussion

We examined the effects of time series length and the number of trials on the accuracy and consistency of FM estimation. Our results show that a sufficient length of data is critical for accurately quantifying orbital stability (figure 3), which is consistent with the results of previous studies [16,37]. This requirement arises because a longer time series better averages out the effects of noise compared to shorter datasets. Ahn *et al*. demonstrated that the bias in FM estimates is not significantly affected by the signal-to-noise ratio and noise characteristics when the sample size is sufficiently large [37]. Generally, statistical guidelines for multiple linear regression recommend a sample size of at least ten times the number of independent variables [38]. Since our model includes 14 independent variables, we expected that approximately 140 consecutive strides would be necessary to ensure reliable FM estimation. Around 140 strides were actually sufficient to establish a strong correlation between FM estimates and the accurate FMs derived from individualized Jacobian matrices, whereas approximately 400 consecutive strides were required to achieve a high level of consistency across experiments when the data were obtained from a single trial (figure 4).

Although a sufficient length of data is important for accurate FM estimation, an excessively long time series alone does not guarantee reliable estimates. OLS regression, commonly used for parameter estimation, provides unbiased estimates under the assumption of uncorrelated noise. However, when temporal dependencies are present in the time series data, such as those observed in autoregressive processes, OLS can introduce systematic bias in parameter estimation [17,39,40]. This bias arises because correlated noise can distort the linear trends within the system’s error dynamics [27]. In the context of gait, stride-to-stride fluctuations are not purely random but exhibit a memory effect; the dynamics of the current stride is influenced by those of previous strides [5,41]. As a result, we observed that the bias in FM estimates did not diminish to zero, even with time series as long as 1000 consecutive strides (figure 3). Additionally, as the data length increases, the influence of individual samples on FM convergence diminishes, and collecting excessive data beyond what is necessary to detect a significant effect may be inefficient and time-consuming. Therefore, segmenting lengthy time series data into multiple appropriately sized intervals can enhance estimation accuracy and reduce the data collection burden in real-world experiments.

Challenges in obtaining accurate FM estimates have been recognized. Hamacher *et al.* reported that while FM demonstrates good discriminatory ability, its reliability is limited, suggesting that variability in FM estimation presents a greater challenge than bias [42]. As shown in the electronic supplementary material, FM estimates occasionally vary across multiple trials even within a participant. Furthermore, the state vector in our analysis comprises lower limb joint angles, which are interdependent due to the coordinated nature of gait kinematics. This interdependence introduces a moderate level of multicollinearity among independent variables (table 1). When multicollinearity is present, the estimated regression coefficients may become sensitive to minor variations in the dataset, leading to unstable parameter estimates in ordinary multiple regression models [14,43,44].

**Table 1.** The average condition number of the five time series of the 500 strides for each subject.

condition number									
**sub1**	**sub2**	**sub3**	**sub4**	**sub5**	**sub6**	**sub7**	**sub8**	**sub9**	**sub10**
11.65	18.97	8.95	13.53	20.41	15.39	14.95	7.07	7.82	8.76
**sub11**	**sub12**	**sub13**	**sub14**	**sub15**	**sub16**	**sub17**	**sub18**	**sub19**	**sub20**
7.88	12.56	9.23	8.17	11.45	11.42	14.39	10.40	21.15	10.14

Despite these challenges, we found that averaging FM estimates across multiple trials significantly improves both the validity and reliability of the estimates. As shown in figure 4, a significant positive interaction is observed between the number of strides and the number of trials, indicating that averaging across multiple trials substantially reduces the number of strides required to achieve acceptable validity and reliability. Since the Jacobian matrix is derived from the average autocorrelation matrix equation (2.3), the averaged FM estimate from multiple trials becomes closer to the accurate value due to the central limit theorem [45]. This indicates that multiple trials could average out the effect of the trial-wise fluctuation of gait stability induced from the non-stationarity of human gait signal, and that segmenting an over-sized time series into proper intervals may further optimize data collection.

Through systematic quantification, we suggest the minimum strides of 140 and 400 for sufficient validity and reliability, respectively, for a single trial case; less strides suffice for the cases of multiple trials (figure 4). However, it is noteworthy that these numbers of necessary strides were obtained when we use 14-dimensional state vectors. Generally, fewer strides are needed to obtain valid and reliable FM estimates when lower-dimensional state vectors are used.

This study, as in multiple previous studies that quantified the orbital stability of human gait using FM [7,12,17], assumes that a typical human gait tends to converge to a unique stable limit cycle. However, human gait patterns are influenced by emotional state, physical health, habitual movement and uncharacterized perturbations [46–49]. Therefore, it might be questionable whether human gait can be properly modelled as a dynamical system converging to a single limit cycle. To examine this common assumption, we analysed the confidence intervals of FM estimates from both experimental and simulation data, finding that they converge after approximately 100 strides (figure 5). This suggests that the dynamics of simulation data, which were synthesized discrete time series with stochastic noise under the assumption of a unique limit cycle, reasonably encapsulate the dynamics of experimental data at least under the current controlled setup with a walking duration of 10 min and fixed speed on a treadmill.

Conducting human experiments in clinical settings typically requires considerable time and effort to collect data samples from participants. Individuals with atypical physical conditions may have limited tolerance for prolonged measurements, and data quality can be compromised by fatigue during the measurement process [50]. The findings of this study emphasize the importance of balancing data length and trial count to achieve both valid and reliable FM estimates while minimizing the data collection burden. These insights can contribute to designing more efficient and effective experiments for assessing orbital stability of human locomotion.

## Data Availability

Data and code associated with this work are available in the Dryad repository [51]. Supplementary material is available online [52].
